# Supplementary material for: Identification of photoperiod-induced specific miRNAs in the adrenal glands of Sunite sheep (Ovis aries)
Source: Front Vet Sci. 2022 Jul 22;9:888207. doi: 10.3389/fvets.2022.888207 (PMC9354845; doi:10.3389/fvets.2022.888207)
Supplement: Supplementary file 1 [file Data_Sheet_1.ZIP › Supplementary file/miRDeep2 file.zip!/miRDeep2 file/Novel_Structure/LP42A2/Novel_557.pdf]

Provisional ID	:	NC_019464.2_15483
Score total	:	400.9
Score for star read(s)	:	3.9
Score for read counts	:	393.2
Score for mfe	:	2.2
Score for randfold	:	1.6
Score for cons. seed	:	
Total read count	:	783
Mature read count	:	777
Loop read count	:	0
Star read count	:	6

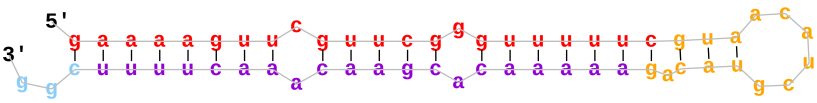

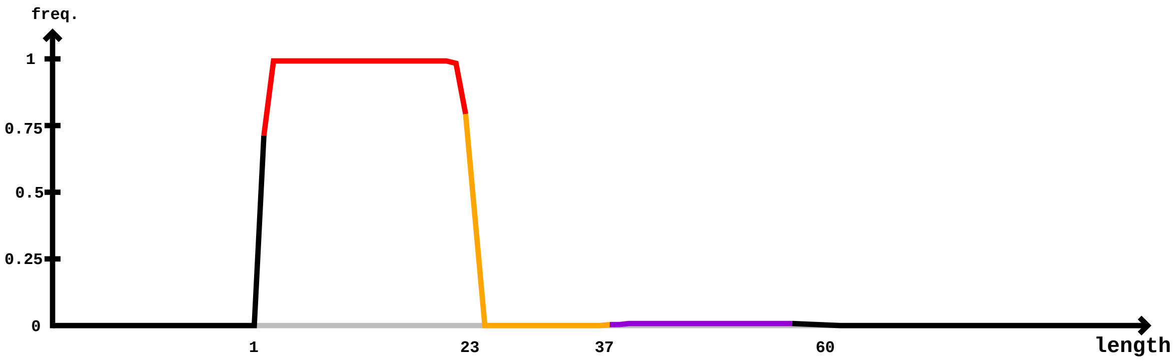

Mature Star

```
5'- auuaucauauuggguuggcugaaaaguucguucggguuuuucguaacaucguacagaaaaacacgaacaaacuuuucggccaacccaguauuucauucaucugcguuuuccu   -3'    obs
    auuaucauauuggguuggcugaaaaguucguucggguuuuucguaacaucguacagaaaaacacgaacaaacuuuucggccaacccaguauuucauucaucugcguuuuccu          exp
    ......((((((((((((((((((((((.(((((.((((((((((......))).))))))).))))).))))))))))))))))))))))).....................    reads   mm   sample
    ....................Aaaaaguucguucggguuuuuc......................................................................    4       1    seq
    ....................gaaaaguucguucggguuuuuc......................................................................    554     0    seq
    .....................aaaaguucguucggguuuu........................................................................    7       0    seq
    .....................aaaaguucguucggguuuuu.......................................................................    130     0    seq
    .....................aaaaguucguucggguuuuC.......................................................................    19      1    seq
    .....................aaaaguucguucggguuuuuc......................................................................    56      0    seq
    .....................aaaaguucguucUgguuuuuc......................................................................    1       1    seq
    .....................aaaaguucguucggguuuuucU.....................................................................    4       1    seq
    .....................aaaaguucguuUggguuuuucg.....................................................................    2       1    seq
    ........................................................aaaaacCcgaacaaacuuuu....................................    3       1    seq
    ..........................................................aaacacgaacaaacuuuu....................................    3       0    seq
```
